# Redox control of vascular biology

**DOI:** 10.1002/biof.1559

**Published:** 2019-09-04

**Authors:** Milan Obradovic, Magbubah Essack, Sonja Zafirovic, Emina Sudar‐Milovanovic, Vladan P. Bajic, Christophe Van Neste, Andreja Trpkovic, Julijana Stanimirovic, Vladimir B. Bajic, Esma R. Isenovic

**Affiliations:** ^1^ Laboratory of Radiobiology and Molecular Genetics Vinca Institute of Nuclear Sciences, University of Belgrade Belgrade Serbia; ^2^ King Abdullah University of Science and Technology (KAUST), Computational Bioscience Research Center (CBRC), Computer, Electrical and Mathematical Sciences and Engineering Division (CEMSE) Thuwal Kingdom of Saudi Arabia

**Keywords:** cardiovascular diseases, cardiovascular system, reactive oxygen species, redox

## Abstract

Redox control is lost when the antioxidant defense system cannot remove abnormally high concentrations of signaling molecules, such as reactive oxygen species (ROS). Chronically elevated levels of ROS cause oxidative stress that may eventually lead to cancer and cardiovascular and neurodegenerative diseases. In this review, we focus on redox effects in the vascular system. We pay close attention to the subcompartments of the vascular system (endothelium, smooth muscle cell layer) and give an overview of how redox changes influence those different compartments. We also review the core aspects of redox biology, cardiovascular physiology, and pathophysiology. Moreover, the topic‐specific knowledgebase DES‐RedoxVasc was used to develop two case studies, one focused on endothelial cells and the other on the vascular smooth muscle cells, as a starting point to possibly extend our knowledge of redox control in vascular biology.

**Abbreviations**
Ang II: angiotensin II
AP‐1: activator protein
CVD: cardiovascular disease
EC: endothelial cells
EGFR: epidermal growth factor receptor
ET‐1: endothelin‐1
H_2_O_2_: hydrogen peroxide
MMP: matrix metalloproteinase
NF‐κB: nuclear factor‐kappaB
NO: nitric oxide
ONOO^−^: peroxynitrite
OxS: oxidative stress
PDGF: platelet‐derived growth factor
PDGFR‐β: platelet‐derived growth factor receptor beta
PTK: protein tyrosine kinases
PTP: protein tyrosine phosphatase
ROS: reactive oxygen species
SHR: spontaneously hypertensive rats
SM‐MHC: smooth muscle‐myosin heavy chain
TF: tissue factor
TF‐FVIIa: tissue factor‐FVIIa
TFPI: tissue factor pathway inhibitor
t‐PA: tissue‐type plasminogen activator
u‐PA: urokinase‐type plasminogen activator
VSMC: vascular smooth muscle cells
XO: xanthine oxidase

## 1 INTRODUCTION

Reactive oxygen species (ROS) and reactive nitrogen species (RNS) stimulate redox homeostasis mechanisms, and thus, they are considered critical signaling molecules for maintaining cellular homeostasis. However, these signaling molecules can be harmful, as well. That is, when ROS/RNS formation outpaces the ROS/RNS removal processes, oxidative stress (OxS) occurs. Chronic OxS leads to several pathologies.^1^, ^2^ In the vascular system, ROS is produced by the vascular smooth muscle cells (VSMCs), endothelial cells (EC), and adventitial cells, among others.^3^ The endothelium regulates the vascular homeostatic system, as it is the barrier that separates blood and tissue. However, when the antioxidant defense system does not remove the excess ROS, it can cause endothelial dysfunction.^4^ This perturbation of the homeostatic functioning of the vascular EC leads to pathologies such as tissue ischemia and atherothrombosis.^5^ ROS is also involved in other pathophysiological processes such as hypertension, inflammation, and vascular remodeling,^6^, ^7^, ^8^, ^9^ which leads to vascular pathologies, including atherosclerosis, arterial hypertension, and cardiovascular diseases (CVDs). Moreover, ROS‐generating systems also facilitate the development of diabetes mellitus, obesity, and hypercholesterolemia, which increases the risk of developing vascular pathologies.^2^

### 1.1 ROS in general

Reactive species are molecules interacting with various biomolecules, including nucleic acids, lipids, and proteins, and generating electron‐deficient species, leading to cell and tissue damages. Different endogenous metabolic and biochemical reactions and exogenous sources, such as ionizing radiation, generate these reactive species. The term “reactive oxygen species” refers to oxygen metabolites, such as superoxide anion radical (O_2_^**•**−^), singlet oxygen (^1^O_2_), hydroxyl radical (OH^**•**^ **─**), and perhydroxyl radical (HO_2_^**•**^), which are yielded by the reduction of the oxygen molecule (O_2_) with two uncoupled electrons.^10^, ^11^, ^12^ Furthermore, ROS with an unpaired electron are free radicals; hence, they are also named oxygen radicals or oxygen‐free radicals,^12^ and free radicals are chemical species that are capable of independent existence with one or more unpaired electron. The unpaired electron transforms the chemical reactivity of the molecule, leading to its increased reactivity compared with the nonradical form of the molecule.^10^, ^13^ Thus, free‐radical forms of ROS can remove electrons from other molecules to gain stability and by that causes new free radical formation, initiating a chain reaction cascade, which consequently impairs the cell/tissue functioning.^14^ In addition, the term ROS cover all oxygen‐containing reactive species, namely, alkoxyl radical (LO^•^), peroxyl radical (LOO⁃), lipid hydroperoxide (LOOH), peroxynitrite anion (ONOO─), hypochlorous acid (HOCl), and ozone (O_3_),^11^, ^12^, ^13^ from which some are without unpaired electrons (hydrogen peroxide (H_2_O_2_), ONOO─, HOCl, and O_3_), and as such, they are not free radicals.^13^

In biological systems, ROS produced by cellular metabolism exert both deleterious, cytotoxic effects, as well as beneficial effects^13^ such as antimicrobial activity,^15^ metabolic pathways regulation,^16^ and cell signaling.^17^ Redox signaling represents pathways in which ROS or some other reactive species serve as messengers to induce cellular responses via redox reactions, implicated in many different physiological and pathological processes in the organism. Thus, ROS can provoke redox modulation of protein kinase cascades and transcription factors leading to different cellular responses, such as cell proliferation and differentiation, altered expression, or synthesis of cytokine and/or adhesion molecules.^12^ Owing to the mostly deleterious nature of ROS, mammals, including humans, have developed a robust antioxidant protection system through the action of enzymes such as Cu─Zn superoxide dismutase (Cu─Zn SOD), catalase, glutathione peroxidase, glutathione reductase, and glutathione‐S‐transferase. Moreover, several diet compounds, particularly from fruits and vegetables, also exhibit antioxidant activities.^12^ An imbalance between ROS generation and removal rate leads to OxS,^12^ and chronic OxS leads to different pathologies, including the vascular pathophysiology.^1^, ^2^

### 1.2 Vascular physiology in general

The vascular system is finely synchronized to adjust the blood flow to the metabolic requirements of the body and is made up of a heart and a closed network of vessels (arteries, veins, and capillaries) that supply oxygen and nutrients to every tissue to maintain cellular homeostasis. Endothelium lines the interior surface of blood vessels—a single layer of homogenous EC layer being in connection with blood and lymph from the circulation and represents a surface (blood/endothelium interface) with roughly 300–1,000 m^2^, whereas the mass is approximately 110 g.^18^, ^19^ VSMC surround the endothelium, establishing the outer layer of arteries. The fibroelastic connective layer and internal elastic lamina located below endothelium provide flexibility and stability for EC. Perivascular cells (pericytes) also surround the EC—multipotential stem cells with the possibility to differentiate into different types of cells among other VSMC, fibroblasts, osteoblasts, connective tissue cells, and adipocytes, providing the EC and VSMC with a stable microenvironment and contractile ability.^20^

EC have a vital function in tissue homeostasis by the regulation of solute and macromolecule transport through the vessels. A glycocalyx layer on the EC surface provides a locally charged barrier to cell and protein movement from the blood through endothelium under physiological conditions.^21^ The endothelium also regulates and maintains vascular tone through interaction with the peripheral nervous system and by synthesizing and releasing vasodilatory factors such as endothelin‐1 (ET‐1), thromboxane, endothelium‐derived hyperpolarizing factor, nitric oxide (NO), and prostacyclin.^22^ Another endothelium function is hemostasis regulation. That is, the endothelium synthesizes compounds, which maintain blood fluidity and are involved in the formation of nonthrombogenic surface and coagulation processes. EC are exposed to lipids (present in the circulation and accumulating in the subendothelial regions) and are also involved in immunological and inflammatory processes, which are associated with atherosclerosis and occlusive vascular disorders.^23^

Furthermore, the endothelium is responsible for the reaction to pathophysiological conditions such as infection or trauma of the neighboring tissues, vessel remodeling, and growth,^24^, ^25^, ^26^ therefore having significant diagnostic and therapeutic potential. EC is capable of synthesizing most of the proteins that constitute the basal lamina and enzymes involved in extracellular matrix remodeling such as matrix metalloproteinase (MMP), which is vital for the blood vessel plasticity and angiogenesis.^27^ Thus, endothelium represents an essential and dynamic endocrine organ due to its different functions as alluded to above.

The fibrinolytic system is a system that restores a blood vessel when the blood clot is not needed, and it includes an inactive proenzyme, the plasminogen, and an active form, plasmin that is responsible for fibrin degradation. The activity of plasminogen activators (PA) such as tissue‐type PA (t‐PA) and urokinase‐type PA (u‐PA) regulates the fibrinolytic potential of the vasculature.^28^ Activation of the EC and the proinflammatory and procoagulant response lead to the synthesis and release of u‐PA.^29^, ^30^ The constitutively produced protein of EC, the PA inhibitor type I (PAI‐1), suppresses t‐PA as well as u‐PA. The activity of PAI‐1 is an independent risk factor for CVD.^31^ The EC synthesized and released 13‐hydroxyoctadecadienoic acid, and the vasodilator factors prostacyclin and NO, which prevents adhesion, aggregation, and activation of platelets.^32^ EC also acts as a natural anticoagulant by expressing the receptor for thrombin, the thrombomodulin, responsible for thrombin conversion into anticoagulant protein from a procoagulant protease. The thrombin bound to thrombomodulin lids to protein C activation, which then binds to the endothelial protein C receptor. Protein C (blood coagulation factor XIV or autoprothrombin IIA) is an anticoagulant serine protease, which participates in coagulation of the blood. Specifically, activated protein C associates with its cofactor, protein S, and inhibit the coagulation process through the inactivation of coagulation factors FVa and FVIIIa.^33^ EC surface has heparin‐like sulfated molecules of glycosaminoglycan, which bind/activate the antithrombin that is the FXa and thrombin leading inhibitor.

Furthermore, the inhibition of the tissue factor‐FVIIa (TF‐FVIIa) complex occurs by its interaction with a specific polypeptide, the tissue factor pathway inhibitor (TFPI), and formation of a stable quaternary complex TFPI‐TF‐FVIIa‐FXa.^34^ Thus, EC prevent blood clotting via a mechanism that involves the heparin‐like molecule thrombomodulin, together with NO and prostacyclin. When an endothelial injury occurs, EC stop the secretion of coagulation and aggregation factors, and synthesize and secrete a large multimeric protein called the von Willebrand factor, which initiates the hemostasis maintenance after injury.^35^ In addition, EC produces the lipid‐mediator platelet‐activating factor, which activates platelets and their attachment to EC.^36^

VSMC represent one of the most frequent types of cells in arteries. VSMC are also essential for the homeostasis of the vasculature, as well as for the vasculature contractions and relaxations, which are responsible for the blood vessel luminal diameter alterations to maintain blood pressure within the normal range. VSMC contractility is controlled regularly by exchanging two different phenotypes.^37^ Different states of VSMC are noted among both the VSMC of the same and various blood vessels. The mature, differentiated VSMC are contractile, and their phenotype is elongated and spindle‐shaped, and these VSMC display low rates of migration and proliferation and express increased levels of proteins that are important for contractility. VSMC shuttle to a dedifferentiated synthetic phenotype with rhomboid shape in certain physiological conditions, including pregnancy or exercise, and after injury of the vasculature, which is vital during vessel remodeling.^38^ Dedifferentiated VSMC are exceptionally proliferative and migratory, and synthesizes extracellular matrix proteins, like elastin and collagen.^39^ Based on all these properties, VSMC are capable of regulating the vessel diameter in short terms and in long terms VSMC are responsible for adaptation by structural remodeling through changing the number of cells and the constitution of connective tissue. The VSMC marker proteins that are most significant in defining VSMC phenotypes include cellular retinol binding protein‐1, smooth muscle myosin heavy chain (SM‐MHC), α‐smooth muscle actin, SMemb/nonmuscle MHC‐B, and smoothelin A and B.^38^ A few of these proteins are structural components of the contractile apparatus or act as the contraction regulators. Generally, the loss of proteins essential to the contractile phenotype indicates the synthetic phenotype. The expression of the two marker proteins, SM‐MHC, and smoothelin, characterizes a mature contractile VSMC phenotype.^40^

## 2 EFFECTS OF ROS

**Figure 1:**
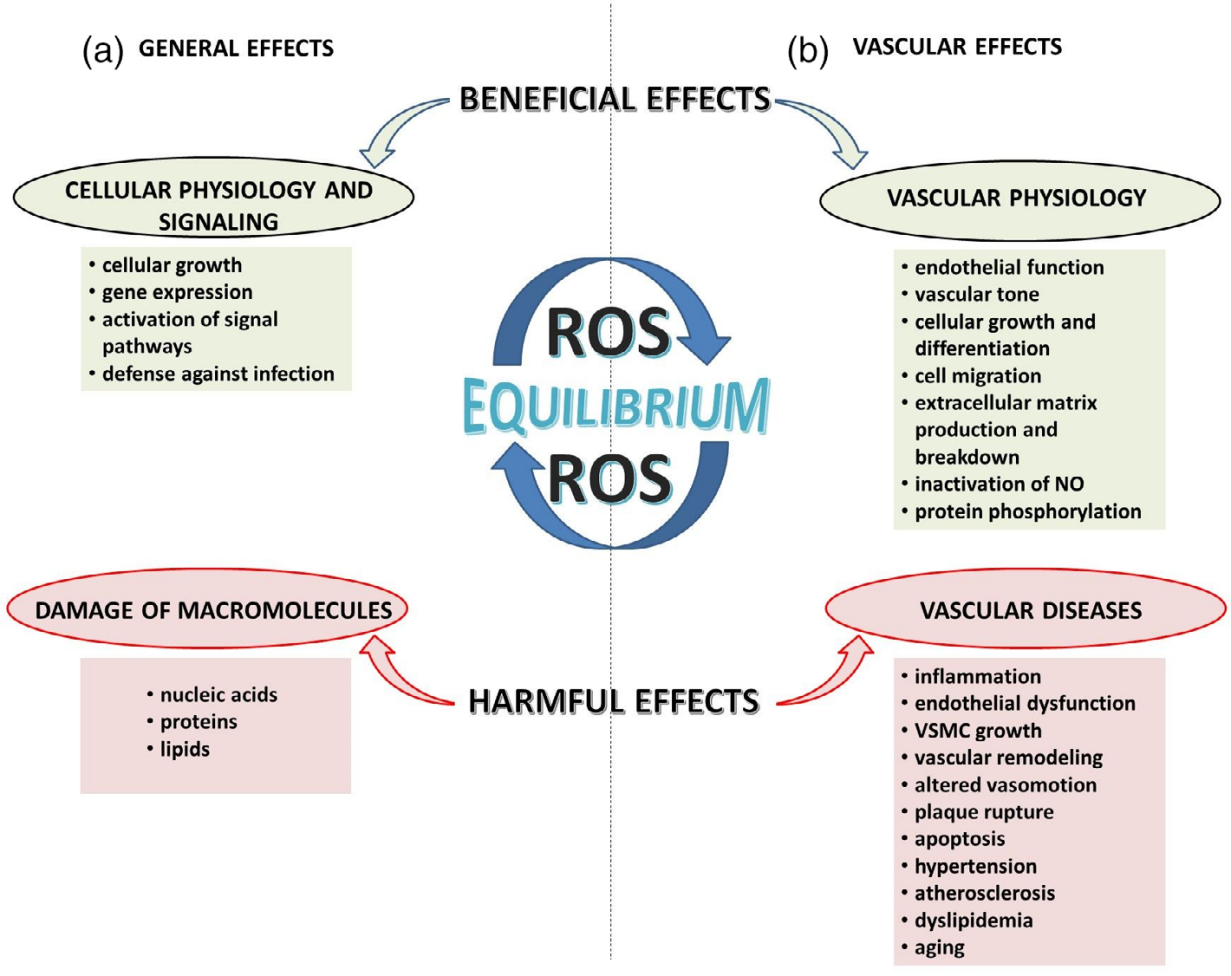
Effects of ROS in physiological and pathophysiological conditions

The human body uses a cellular antioxidant defense system to neutralize free radicals and, consequently, OxS. In OxS, ROS are involved directly or indirectly in the macromolecules deterioration, along with oxidative deterioration of nucleic acids, proteins, and lipids.^41^ However, ample evidence suggests that ROS directly activate OxS‐responsive pathways regulating different cellular processes, additionally leading to the progression of diseases. OxS is associated with carcinogenesis,^42^ neurodegeneration,^43^ atherosclerosis, diabetes,^3^ and aging.^44^ ROS are also critical physiological modulators of the redox state signaling molecules.^45^ ROS changes the function of the protein by the mechanism that includes the modifications of cysteine (Cys) residues, which are redox‐reactive. Cys residue oxidation promotes the formation of reactive sulfenic acid (─SOH) that can interact with nearby Cys‐forming disulfide (─S─S─) bonds or endure additional oxidation to sulfinic (─SO_2_H) or sulfonic (─SO_3_H) acid.^46^ These modifications (except sulfonic acid and to a lower extent sulfinic acid) reverse specific reducing systems including peroxiredoxin and thioredoxin, suggesting that these modifications are involved in redox sensing and signaling and regulation of protein structure/function.^47^ Numerous studies show that ROS influence many different signaling pathways involving molecules, such as tyrosine and Rho kinases, mitogen‐activated protein (MAP) kinases, transcription factors (HIF‐1, nuclear factor‐kappaB [NF‐κB], and activator protein‐1 [AP‐1]), as well as protein tyrosine phosphatases (PTPs) involved in cardiovascular, neural, and renal cell function.^48^, ^49^ ROS elevates the concentration of intracellular free Ca^2+^ ([Ca^2+^]_i_) by the activation of ion channels, and ROS upregulates the expression of proinflammatory and proto‐oncogene genes (Figure 1).^50^

## 3 REDOX SIGNALING IN THE VASCULAR PATHOPHYSIOLOGY

In vascular tissue, diverse systems generating ROS interrelate to establish a combined redox modulation.^51^ Nonetheless, the majority of tissue damage and pathology is a result of excessive ROS production, which leads to inflammation in the vascular tissues, while excessively low levels of ROS interrupt the oxidant physiological role in vasodilation and cellular growth.^52^ Furthermore, it has been suggested that “intermediate” ROS levels could facilitate the physiological response in the vasculature, while chronically elevated ROS level characteristic for OxS could be associated with CVD.^53^ Hence, ROS participate in the pathophysiology of the vascular disease, but also regulate vascular function in healthy vessels^54^ (Table 1). In addition, ROS is part of an adaptive response in vascular diseases.^87^ In Table 2, we summarized some of the effects of ROS on vascular physiology in animal and human studies. These distinctive roles of ROS may be a consequence of the different physicochemical properties of ROS. For example, H_2_O_2_ biological half‐life is expanded compared with the half‐life of O_2_^−^ and OH^−^ and can diffuse through lipid membranes, and the charge of the O_2_^−^ molecule prevents it from crossing the cell membranes except for the possibility to cross the membrane through ion channels.^3^ Besides, reductive stress (RS) is another deleterious factor that disturbs the redox state and promotes vascular pathophysiology.^103^, ^104^, ^105^ Furthermore, RS increases s‐glutathionylation of proteins, and that may be involved in uncoupling of eNOS that elevates O_2_^−^ production and induces OxS.^103^, ^104^, ^105^, ^106^

**Table 1:** Some ROS species important for vascular physiology and pathophysiology

ROS	Mechanism of generation/enzymatic source	Physiological concentrations	Reference	Elevated concentrations	Reference
•O_2_	The electron transport chain of mitochondria	EC growth, proliferation, survival, and angiogenesis	55	Enhanced vasoconstriction Impaired vasorelaxation Decreased sGC activity/expression Vascular remodeling Sympathetic activation Endothelial dysfunction Peripheral resistance DNA damage EC apoptosis VSMC hypertrophy VSMC matrix regulation Hypertensions Atherosclerosis	56, 57 57 57 58, 59, 60, 61 57 57, 58 57 57 62 62 62 58, 60, 63, 64 58, 65
	Monooxygenase	Contraction–relaxation			
	NADPH oxidase	Maintaining cardiac and vascular integrity	56		
	Xanthine oxidase Lipooxygenase eNOS uncoupling		58		
H_2_O_2_	From •O_2_ by SOD	EC growth, proliferation, survival, and angiogenesis Endothelium‐dependent vasorelaxation Cytoskeletal reorganization of EC Inflammatory responses of EC Endothelium‐regulated vascular remodeling Vasodilation Synthesis/release of endothelium‐derived relaxing factor	55, 66	Impaired proliferation and/or decreased viability of EC EC apoptosis VSMC hypertrophy VSMC proliferation VSMC matrix regulation VSMC apoptosis Vascular relaxation and hyperpolarization Hypertension Atherosclerosis	55, 67 62, 68 55, 62, 69 62 62 62 54, 70, 71 62 62
	Directly produced by:		55		
	Glucose oxidase		55		
	Xanthine oxidase		55		
	‐NADPH oxidase 4 isoform		55		
			55, 72, 73, 74, 75, 76		
			77		
•OH	Produced in Haber–Weiss reaction from •O_2_ and H_2_O_2_ from reactions with hypochlorous acid	Vasodilation VSMC relaxation Synthesis/release of endothelium‐derived relaxing	77 77 77	Atherosclerosis Lipid peroxidation	78 79
ONOO^−^	Produced in reaction of NO and •O_2_	Regulation of vascular contraction/relaxation Trigger stress adaptation Protection of vascular endothelium	80 81 82	Impairment of vascular relaxation by various mechanisms EC and VSMC apoptosis Peripheral vascular failure Lipid peroxidation Oxidation of BH_4_ Oxidation of sGC Oxidative DNA damage Endothelial dysfunction	83, 84 84, 85, 86 84 57 57, 84 57 84 84

*Note*: Under physiological conditions, ROS in the vasculature are produced in a controlled manner at low concentrations and function as signaling molecules. Increased ROS production leads to pathological conditions of vascular system.

Abbreviations: •OH, hydroxyl radical; BH4, tetrahydrobiopterin; EC, endothelial cell; eNOS, endothelial nitric oxide synthase; H_2_O_2,_ hydrogen peroxide; NADPH, nicotinamide adenine dinucleotide phosphate; NO, nitric oxide; O_2_, superoxide anion; ONOO^−^, peroxynitrite; sGC, soluble guanylate cyclase; SOD, superoxide dismutase; VSMC, vascular smooth muscle cells.

**Table 2:** The effects of ROS on vascular physiology‐ animal and human studies

Effects of ROS	Condition and/or treatment		Species	Ref.	
Endothelial dysfunction	↑ OxS ↑ XO activity	Ang II‐induced vascular dysfunction	dTGR SDR	88
↑ Arteriolar tone	↑ XO activity		SHR	89
Correct hypertrophy of renal and cardiac tissue	↑ XO activity	Allopurinol Sodium restriction	SHR DSS rats	90, 91
↑ Systolic blood pressure Endothelial dysfunction Vascular hypertrophy	↑ O_2_^−^ ↑ p22^phox^ mRNA	Irbesartan	SHR WKY	92
Atherosclerosis	↑ ROS	Ang II‐mediated MAP kinase activation	Rat VSMC	93
↑ Systolic blood pressure	↑ O_2_^−^	Ang II‐induced hypertension		94
Stimulate vascular smooth muscle cell growth	↑H_2_O_2_ ↑O_2_^−^	Naphthoquinolinedione LY83583		95
↓ Expression of proinflammatory molecules	↑ O_2_^−^	Angiotensin receptor inhibition gp^91phox^ NAD(P)H oxidase inhibiton	Hypertensive DSS rat	58
↓ Vascular superoxide production	↑ superoxide production ↑ ET‐1	ETA receptor inhibition	DOCA‐salt hypertensive mice and rats	96, 97
Attenuated blood pressure elevation	↑ O_2_^−^	Ang II–induced hypertension Inhibited association of p47^*phox*^ and gp91^*phox*^	Mice/ Aortic rings	46
↑ Mean blood pressure Nox1(+/Y) ↓ Production of superoxide in Nox1(−/Y) Preserved endothelium‐dependent relaxation in Nox1(−/Y)	↑ ROS	Ang II‐induced hypertension	Nox1‐deficient (−/Y) mice	98
↑ Systolic blood pressure ↑ Aortic hypertrophy Development of cardiovascular pathologies	↑ superoxide production		VSMC TgSMCnox1	99
No changes in blood pressure of p47^phox−/−^deficient mice after Ang II tratment	↑ O_2_^−^		p47^phox−/−^deficient mice	63
↓ Aneurysm formation ↓ NO_*x*_ levels	↑ ROS	NADPH oxidase inhibition	iNOS^−/−^ mice	100
Dysfunction of the endothelium Vascular remodeling	‐	ET‐1 overexpression	C57BL/6 TG and WT mice	101
↓ NO levels	↑ O_2_^−^ production	PMA stimulation	Hypertensive and normotensive patients	102
↑Oxidized/reduced glutathione ratio ↓ Activity of superoxide dismutase, catalase, and glutathione peroxidase Development of cardiovascular complications	↑ ROS	‐	Hypertensive and normotensive patients	53

Abbreviations: ↑, increased; ↓ decreased; Ang II, angiotensin II; DOCA, salt‐deoxycorticosterone acetate‐salt; DSS, Dahl salt‐sensitive; dTGR, double‐transgenic rats; ET‐1, endothelin‐1; iNOS−/−, iNOS‐deficient; ONOO^−^, peroxynitrite; OxS, oxidative stress; PMA, phorbol myristate acetate; SDR, Sprague Dawley rats; SHR, spontaneously hypertensive rats; TG, transgenic; TgSMCnox1, transgenic mice overexpressing Nox1 in smooth muscle cells; VSMC, vascular smooth muscle cell; WKY, Wistar‐Kyoto rats; WT, wild type (nontransgenic); XO, xanthine oxidase.

### 3.1 Xanthine and NAD(P)H oxidase as the culprit of ROS in vascular pathophysiology

The most prominent origins of ROS in the vascular tissue are xanthine oxidase (XO) and NAD(P)H oxidase. NAD(P)H oxidase regulates the conversion of xanthine dehydrogenase (XDH) to XO. The modulation of XDH to XO reduces oxygen to O_2_^−^ and H_2_O_2_. XO regulates the oxidation process of hypoxanthine and xanthine and generates O_2_^−^ in the vascular endothelium.^3^, ^107^

The increase in XO activity has been associated with endothelial dysfunction in rats overexpressing renin and angiotensinogen from implanted human genes.^88^ On the other hand, in spontaneously hypertensive rats (SHR), increased activity of XO was found in the microcirculation of the mesentery.^89^ The increased XO activity has also been found in the renal tissue of SHR and Dahl salt‐sensitive (DSS) rats, while the discovery that allopurinol can correct the hypertrophy of renal and cardiac tissue in SHR without lowering the blood pressure implies the involvement of XO in organ damage induced by elevated blood pressure.^90^, ^91^ Models for ischemia–reperfusion injury and heart failure also demonstrated the involvement of XO.^3^

NAD(P)H oxidase is an enzyme complex that uses NADH/NADPH as the donor of an electron in the reduction of O_2_ and consequent formation of superoxide. The membrane‐bound p22^phox^ subunit is crucial for the NAD(P)H oxidase complex activation and is accompanied by an elevated production of O_2_^−^ in humans with coronary artery disease.^108^ The increased levels of OxS byproducts, together with elevated oxidative DNA damage, were found in samples obtained from patients with arterial hypertension.^53^

The mononuclear cells isolated from the blood of patients with essential hypertension exhibited increased O_2_^−^ production after treatment with angiotensin II (Ang II) or ET‐1 when compared with controls.^102^ The majority of studies investigating NAD(P)H oxidase activation in vascular cells focused on Ang II signaling, which implicates receptor tyrosine kinases, protein kinase C, c‐Src, and phospholipase D.^109^ The expression/activity of NAD(P)H oxidase was found to be increased in rats with hypertension caused by Ang II treatment.^94^ A NAD(P)H oxidase inhibitor, decreasing both vascular O2^−^ synthesis and hypertension in mice, supports this finding.^110^ The NAD(P)H oxidase Nox1 isoform participates in the Ang II‐induced hypertensive response. The reduced production of O_2_^−^ and the blunted effect of Ang II on the blood pressure were both found in Nox1‐deficient mice.^98^

On the other hand, the overexpression of Nox1 enhances Ang II‐induced O_2_^−^ formation, VSMC hypertrophy, and hypertension in transgenic mice.^99^ Furthermore, in cytosolic NAD(P)H p47^phox^ subunit‐deficient mice, the endothelial dysfunction, O_2_^−^ production, and Ang II‐induced hypertensive response were blunted.^63^ Moreover, NAD(P)H oxidase was associated with raised O_2_^−^ production in the arterial tissue of SHR.^92^

The produced ROS from NAD(P)H can react with products of nitric oxide synthase (NOS), which may further amplify vascular function. For example, O_2_^−^ generated from NAD(P)H oxidase reacts with NO, forming peroxynitrite (ONOO^−^), which consequently induces changes in the NOS enzyme subunits resulting in further O_2_^−^ formation and the declination of NO bioavailability, which impairs endothelium‐dependent relaxation in a model of salt‐induced hypertensive rats.^111^ ROS synthesized by NAD(P)H oxidase is required for iNOS expression in microvascular EC.^112^

The membrane‐bound gp^91phox^‐containing NAD(P)H oxidase is identified as a source of excessive O_2_^−^ linked with elevated activity of the renin–angiotensin system as shown in renovascular^113^ and salt‐induced hypertension^114^ models. Treatments with the angiotensin receptor blocker and with the gp^91phox^ NAD(P)H oxidase inhibitor restrained the elevated O_2_^−^ aortic generation and the expression of the proinflammatory molecules in a model of hypertensive DSS rats. Importantly, ROS‐induced injury was shown to lead to further ROS formation while proinflammatory cytokines activated the system of NAD(P)H oxidase.^58^ The JAK/STAT cascade, which regulates the transcription of proinflammatory genes, is also upregulated by ROS synthesized by the Ang II and platelet‐derived growth factor (PDGF) activity. Inhibition of the NAD(P)H oxidase subunit p47^phox^ inhibits Ang II‐induced activation of JAK/STAT signaling and interleukin‐6, which suggests that the NAD(P)H oxidase activity generates the ROS involved in this pathway.^115^

Furthermore, ROS produced at one subcellular locus can initiate ROS synthesis in another locus by signal transmission. For example, mitochondria‐derived ROS production activates ROS formation by NAD(P)H oxidase.^116^ The NAD(P)H oxidase increased activity in pulmonary microvascular EC produces extracellular O_2_^−^. The extracellular O_2_^−^ moves through chloride channel 3 into cells, where it triggers Ca2^+^ mobilization and mitochondrial O_2_^−^ production. Thus, ROS generated from endothelial NAD(P)H oxidase mediates intracellular signaling.^117^, ^118^

The ET‐1 also induces an increase in ROS generation by the activation of NAD(P)H oxidase pathway. ET‐1 via ETA receptor activation increases O_2_^−^ production in the DOCA‐salt hypertensive rat model, while the ETA receptor inhibition decreases the production of vascular superoxide.^96^, ^97^ Moreover, endothelium‐restricted human ET‐1 overexpression was shown to cause dysfunction of the endothelium and vascular remodeling in mice through the NAD(P)H oxidase pathway.^101^ Besides, NAD(P)H oxidase pathway, dysfunction of endothelium, and vascular remodeling can be the result of an activated extracellular signal–regulated kinases (ERK) signaling pathway. Numerous studies have shown that increased level of oxLDL induces accumulation of ROS, which stimulates ERK phosphorylation, upregulates the expression of endothelial transcriptional factor AP‐1 and ET‐1, and, consequently, promotes the progression of atherosclerosis.^119^, ^120^, ^121^

Activation of NAD(P)H oxidase is also involved in the pathogenesis of abdominal aortic aneurysm. That is, samples obtained from subjects suffering from aneurysms of the abdominal aorta exhibit overexpressed NADPH oxidase and raised level of O_2_^−^ in the endothelium and within the aortic wall. ROS synthesized by the NAD(P)H oxidase activity facilitate the inflammatory process in the aortic endothelium and stimulate the extracellular matrix impairment through the MMP‐2 activation and apoptosis of VSMC. A murine aneurysm model was used to show that inhibition of ROS mitigates the formation of the aneurysm.^100^ Also, the cyclic stretch of VSMC stimulates the expression of MMP‐2. This response is blunted in murine cells deficient in the p47^phox^ subunit of NAD(P)H oxidase, thus implying the function of ROS generated by the NAD(P)H oxidase in MMP‐2 signaling in VSMC.^122^ Moreover, Ang II induces MMP‐2 via the p47^phox^ subunit.^123^ Also, ROS generated by macrophage‐derived foam cells modifies the activity of MMP‐2 and MMP‐9 in vitro.^124^

Various growth factors (such as PDGF and transforming growth factor β) and hormones are involved in the modulation of NAD(P)H oxidase activity/expression, while ROS regulates the activity of many signaling enzymes (tyrosine and MAP kinases, PTPs) in the vasculature.^3^ Redox signaling also mediates the activity of transcription factors in the vascular cells. In addition, the laminar flow and the shear stress influence the ROS activity in both physiological and pathophysiological settings.

### 3.2 The effects of ROS on tyrosine and MAP kinases, and PTPs in vascular pathophysiology

ROS regulates the tyrosine kinases (receptor and nonreceptor forms) in the vasculature. Receptor tyrosine kinases, PDGF receptor beta (PDGFR‐β), and the epidermal growth factor receptor (EGFR) require ligand‐stimulated signal transduction. It appears that H_2_O_2_ generation is mandatory for signal transmission through PDGFR‐β.^125^, ^126^ Furthermore, Ang II stimulates EGFR and PDGFR‐β through transactivation. The nonreceptor tyrosine kinase Src also contributes to the H_2_O_2_‐dependent Ang II‐stimulated EGFR transactivation.^127^ Activation of tyrosine kinases regulates NAD(P)H oxidase, which additionally amplifies ROS production in the vascular tissue. The transactivation of EGFR and PDGFR‐β stimulates MAP kinases in VSMC.^128^

PDGF and Ang II activate MAP kinases in the vascular tissue.^129^ H_2_O_2_ and O_2_^−^ activate MAP kinases in VSMC.^95^ H_2_O_2_ stimulates p38 MAP kinase, required for redox‐sensitive signal transduction initiated by Ang II in VSMC.^130^ ROS implicated in MAP kinase activation induced by Ang II has also been derived from NAD(P)H oxidase in VSMC.^93^

Protein tyrosine phosphorylation is also affected by ROS. Protein tyrosine kinases (PTK) and PTPs regulate tyrosine phosphorylation. The complex of PTP enzymes dephosphorylates PTK substrate proteins and counteracts PTK activity. Both receptor and nonreceptor PTPs are susceptible to O_2_^−^ and H_2_O_2_. Exposure of cells to ROS inactivates PTP through oxidation of Cys residue in its structure and increases protein phosphorylation.^131^ However, this process is reversible, and PTPs is present in two states, either with a reduced or with oxidized Cys. Ang II may influence the oxidation of PTP‐activated NAD(P)H oxidase and O_2_^−^ generation.^132^, ^133^ Also, Ang II stimulates the expression of the vascular cell adhesion molecule‐1 via NF‐κB activation through ROS signaling.^134^

### 3.3 ROS‐induced activation of transcription factors in vascular pathophysiology

The activation of ROS by NF‐κB occurs through the activation of inhibitory κB kinases. The NF‐κB signaling modulates the expression of genes involved in inflammation and stimulates the ROS generation. In diabetic mice, the activation of NF‐κB increased the generation of the proinflammatory cytokine, the tumor necrosis factor‐alpha, which in turn elevated O_2_^−^ production by NAD(P)H oxidase.^135^ Ang‐II stimulated endothelial ROS generation through the stimulation of NF‐κB signaling pathway in DSS rats.^136^ One of the sources of ROS (O_2_^−^ and H_2_O_2_) generated through NF‐κB signaling is NAD(P)H oxidase. The NF‐κB cis‐acting elements regulate the promoter of p22^phox^NAD(P)H oxidase gene in human aortic smooth muscle cells.^137^ The findings that revealed that NF‐κB enters the mitochondria of the obese mouse with diabetes and increases mitochondrial O_2_^−^ suggest the nontranscriptional role of NF‐κB in ROS production.^138^ Since NF‐κB activity leads to elevated ROS production, while ROS activates NF‐κB, the ROS generated by NAD(P)H oxidase will upregulate NF‐κB, initiating a positive feedback mechanism for ROS generation.^108^

## 4 HEMODYNAMICS AND REDOX SIGNALING

Redox signaling likely also influences the mechanical forces acting on the vascular wall. That is, elevated intraluminal flow stimulates the generation of O_2_^−^ and H_2_O_2_ in intact blood vessels. In VSMCs, shear stress was demonstrated to induce ROS generation.^139^ The ROS activity in cultured EC during oscillatory shear stress is derived from the activation of XO. The attenuation of O_2_^−^ production in the vasculature affected by oscillatory shear was found in cells lacking NAD(P)H oxidase activity, or by application of oxypurinol, a specific blocker of XO.^107^ However, the exact mechanism is still undetermined and may implicate NAD(P)H, and XO and/or mitochondrial enzymes.^132^ Laminar shear upregulates the expression of the extracellular SOD and cytosolic copper and zinc SOD in human aortic EC, and may regulate protective vascular response.^140^ Finally, ROS, particularly O_2_^−^ and H_2_O_2_, can diminish the efficacy of the antioxidant system, leading to the promotion of OxS. These signaling molecules play a significant role in the pathophysiology of the vasculature.

## 5 DEVELOPING LEADS TO EXTEND OUR UNDERSTANDING OF REDOX CONTROL OF VASCULAR BIOLOGY

Data‐mining and text‐mining techniques have been used to explore the information contained in published biomedical literature. Advancements in these techniques have led to the development of several topic‐specific knowledgebases (KB),^141^, ^142^, ^143^, ^144^, ^145^, ^146^, ^147^, ^148^, ^149^, ^150^, ^151^, ^152^, ^153^, ^154^, ^155^, ^156^, ^157^, ^158^, ^159^ including the first topic‐specific KB for redox control of vascular systems, named DES‐RedoxVasc.^160^ DES‐RedoxVasc was constructed using the search query: (human OR mouse OR rat OR mammal*) AND (radical* OR peroxide* OR “reductive stress” OR ROS OR “reactive oxygen species” OR RNS OR “reactive nitrogen species” OR redox OR “reduction–oxidation reaction” OR oxidative OR nitrosative OR peroxide* OR superoxide* OR detoxifi* OR antioxid* OR “polyunsaturated fatty acids” OR “arachidonic acid” OR “linoleic acid” OR hydroperoxide* OR “hypochlorous acid” OR peroxynitrit* flavoprot* OR xanthine oxidase* OR “cytochromes P450” OR catalase* OR sulfiredoxin* OR peroxiredoxin*) AND (“angina pectoris” OR anemia OR aneurysm* OR angio* OR arter* OR atrial OR atrioventricular OR aort* OR bradycardia OR blood OR brain OR circulati* OR clogging OR cardio* OR coronary OR edema OR heart OR ischemic OR hemo* OR hypertension OR leukemia OR leuko* OR macroangiopathy OR microangiopathy OR neovascularization OR occlusion OR pericardi* OR sepsis OR “sickle cell” OR tachycardia OR tachyarrhythmia OR thromb* OR vaso OR vein* OR ventricular OR vascular* OR vessel*) to retrieve all literature specifically focused on research related to redox effects on the cardiovascular system in mammalian organisms. This allowed for the retrieval and analyses of published information from 233′399 PubMed (based on article abstracts) and PubMed Central documents (based on the complete text in the article) linked to redox processes in the cardiovascular system. Users can easily explore the analyzed documents in DES‐RedoxVasc through links between various concepts from 28 topic‐specific dictionaries such as pathways, diseases, genes/proteins, miRNAs, toxins, drugs, biological processes, molecular functions, and so on. DES‐RedoxVasc can be used to search for hypotheses and potentially new knowledge in vascular biology. A published example in 160 shows that the semantic similarity tool in this KB linked ZFAS1 (long non‐coding RNA) and miR‐27b, even though there is no literature linking the functioning of miR‐27b and ZFAS1. Nonetheless, miR‐27b and ZFAS1 are linked to different vascular pathologies, and the DIANA tool, LncBase Predicted v.2,^161^ predicts that ZFAS1 binds hsa‐miR‐27b‐3p, which supports the possibility that the link suggested by DES‐RedoxVasc may be correct. The possible role of ZFAS1 in the fine tuning of levels of miR‐27B, a microRNA that is known to be responsive to OxS, has not been explored.^160^ As a consequence, we used DES‐RedoxVasc to develop additional case studies.

**Figure 2:**
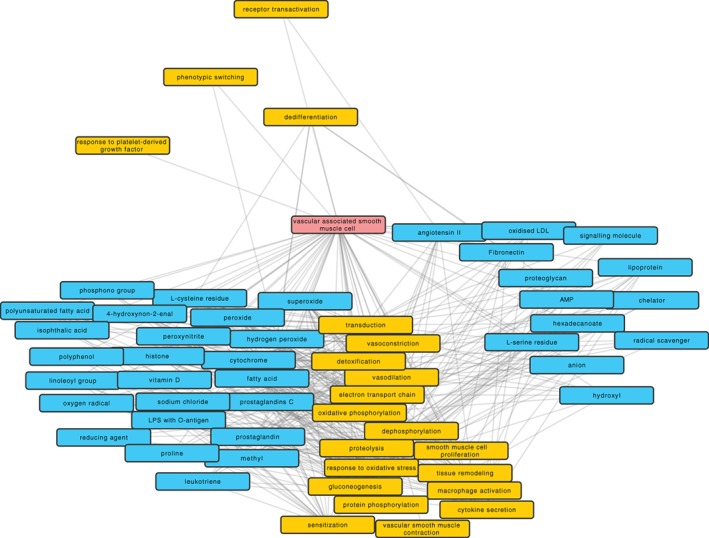
Chemical substances connecting vascular smooth muscle cells (VSMC) and gene ontology (GO) biological functions. Chemical substances from ChEBI are shown with a blue background color and GO biological functions with yellow color. The transparency of the edge indicates the frequency of co‐occurrence of the connected terms. The graph was formed by extracting all ChEBI or GO term co‐occurrences with “vascular associated smooth muscle cell” from the DES‐RedoxVasc KB. The connection between CheBI and GO terms already extracted in the first step was also added. A general filter was applied to the edges to have at least two articles reporting co‐occurrence. The layout of the graph was first force‐directed by the amount of co‐occurrence between two terms, but then manually adjusted for readability of the nodes

Case Study 1: Here, we characterize the biological functions of VSMC relative to chemical substances in the literature in connection with both VSMC and gene ontology biological functions that were associated with VSMC. As such, Figure 2 gives an overview of substances that are vital for homeostasis or can disturb the proper functioning of VMSC

The following redox‐related molecules discussed in this manuscript were retrieved: “oxygen radical,” “superoxide,” and “hydrogen peroxide.” However, DES‐RedoxVasc points out additional molecules that should be explored (via https://www.cbrc.kaust.edu.sa/des-rv/) in this context. For instance, it would be interesting to look at the relationship between “H_2_O_2_” and “Ang II,” as Ang II and other chemotactic factors are dependent on H_2_O_2_ for their release.^162^ It is therefore of interest knowing which GO terms are linked to both H_2_O_2_ and Ang II. In the DES‐RedoxVasc KB, they are linked to vasodilation, vasoconstriction, macrophage activation, as well as VSMC that were the starting point of this exploration.

**Figure 3:**
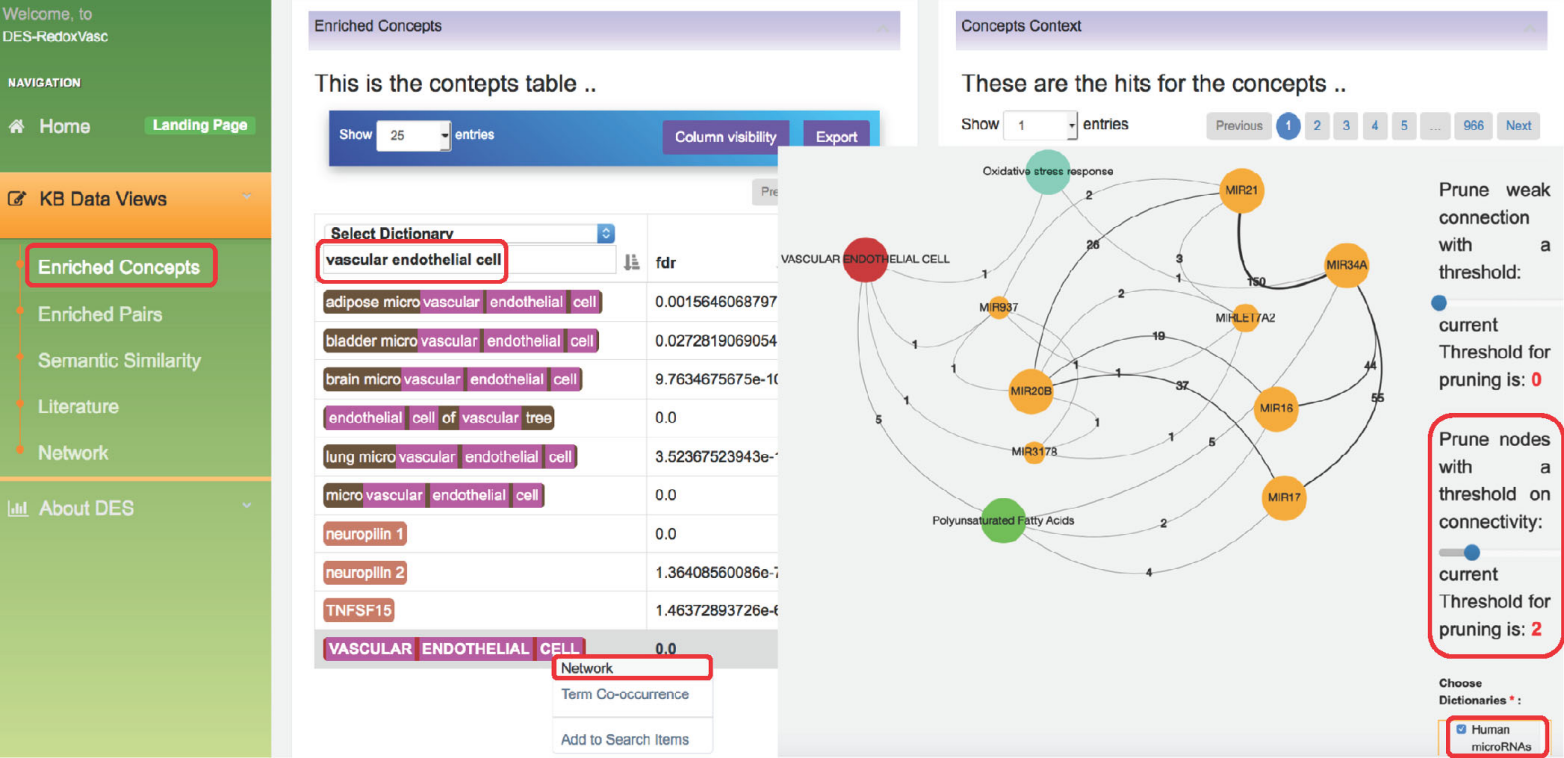
DES‐RedoxVasc network illustrating microRNAs that may affect the relationship among “VASCULAR ENDOTHELIAL CELL”, “Oxidative stress response”, and “Polyunsaturated Fatty Acids.” The orange circles denote concepts from the “human microRNAs” dictionary; the green circles denote concepts from the “HFO Ontology (Bioportal) Heart Failure Ontology” dictionary; the blue circles denote concepts from the “Pathways (KEGG, Reactome, UniPathway, Panter)” dictionary; and the red circles denote concepts from the “Human Anatomy” dictionary

Case Study 2: Studies have demonstrated that both dietary PUFA and fish oils exhibit a protective role in CVD.^163^, ^164^, ^165^, ^166^ PUFAs provide several benefits including modulating lipid metabolism, reducing the production of inflammatory cytokine, and facilitating improvements in vascular EC function.^167^ PUFAs also transiently increase the levels of ROS that activate the OxS‐response transcription factor NFE2L2/NRF2 (nuclear factor, erythroid derived 2, like 2) in pigment epithelial cells from the human retina,^168^ which transcribe several antioxidant genes. PUFAs were also shown to modulate noncoding RNAs^169^, ^170^ that may be the mechanism used to mediate the chemoprotective and antioncogenic properties of PUFAs. Thus, we here also used DES‐RedoxVasc to explore the microRNAs linked to vascular ECs, OxS response, and n‐3 polyunsaturated fatty acids (PUFA) (see Figure 3). We start this exploration by clicking the “Enriched Concepts” link in DES‐RedoxVasc. On this page, we used the search bar to filter the concept “vascular endothelial cell,” and then used this concept right‐click menu to generate a network (see Figure 3). On the “Network” page, we selected four dictionaries including “HFO Ontology (Bioportal) Heart Failure Ontology,” “Pathways (KEGG, Reactome, UniPathway, Panter),” and “Human microRNAs” dictionaries. Then, the “vascular endothelial cell” node was highlighted to expand the associated concepts with links from the selected dictionaries. Then, we only selected the “Human microRNAs” dictionary (as microRNAs linked to vascular ECs, OxS response, and PUFAs are the focus of this case study), and highlighted the “Oxidative stress response” node to expand/link the associated concepts from the “Human microRNAs” dictionary. We repeated this step for other nodes including the “Polyunsaturated Fatty Acids,” “MIR3178,” and “MIR937.” All additional nodes with two or more edges (“MIR34A,” “MIR20B,” and “MIR7A2”), generated in step 3, were also expanded/linked with their associated concepts in the “Human microRNAs” dictionary. We then pruned the nodes using the connectivity threshold of 2 (see Figure 3).

Several of the noncoding RNAs (“MIR15B” [miR‐15b], “MIR16” [miR‐16], “MIR17” [miR‐17], and “MIR20B” [miR‐20b]) retrieved by DES‐RedoxVasc are modulated by PUFAs. That is, three different PUFA‐treated glioblastoma cell lines consistently exhibited an increase in the levels of miR‐20b, and decreased levels of microRNA (miR‐16 and miR‐17) that induces apoptosis‐specific expression changes.^169^ In addition, the level of miR‐15b increases in rats injected with the colon carcinogen and azoxymethane, and fed by fish oil/n‐3 PUFA rich diets.^171^

This report by 171 is interesting as the generation of mitochondrial ROS is promoted by miR‐15b, as well as mitochondrial dysfunction, through the inhibition of SIRT4 (exclusively localized in mitochondria).^172^ SIRT4 is also associated with photoaged skin and stress‐induced cellular senescence, which linked senescence‐associated mitochondrial dysfunction to both miR‐15b and SIRT4.^173^ Moreover, miR‐20b upregulated by PUFAs directly targets AKT3, and AKT3 silencing decreases the levels of VEGF.^174^ Specifically, in primary ECs, VEGF stimulation and the downstream mitochondrial biogenesis process required AKT3, and the blockade of AKT3 also reduces PGC‐1α‐dependent gene expression.^175^ This suggests that even though mitochondrial biogenesis is tightly interlinked to antioxidant systems, both miR‐15b and miR‐20b prevent mitochondrial functioning under unregulated OxS conditions. This decrease in mitochondrial biogenesis substantially decreases dysfunctional mitochondria produced by excessive ROS, which can be viewed as a quality‐control process.

Figure 3 further shows that both the “MIR20B” and “VASCULAR ENDOTHELIAL CELL” nodes are further linked to two additional microRNAs, “MIR3178” (miR‐3,178) and “MIR937” (miR‐937), which suggest that they may function in the same or a closely linked mechanism. For now, we know that the levels of miR‐20b, miR‐937, and miR‐3,178 are upregulated in a human vascular EC line (EAhy926) infected with the DENV‐2 (TR1751 strain),^176^ which may be a consequence of a mechanism counteracting the DENV infection‐induced OxS.^177^

## 6 CONCLUDING REMARKS

Endothelial ROS production has a prominent role in regulating the vascular redox homeostatic mechanisms in the vascular system. By understanding the ROS/RNS removal processes, we may influence the overall perturbing homeostatic functioning of the vascular endothelial system and the underlining consequence of a disbalance portraying as OxS in which it elevates inflammation and vascular remolding, activating the events for CVD.

The most prevailing players in vascular ROS pathology are XO and NAD(P)H oxidase, with links to various models of heart failure, ischemia injury, and DNA damage in patients with arterial hypertension, and so on. Interestingly, NAD(P)H was found to be in an ROS MMP‐2 signaling pathway in VSMC, suggesting some interwoven pathways of redox signaling, PTK and phosphatase activation, growth factors, vasoactive hormones, and transcription factors. To broaden our understanding of the redox control of the vascular endothelial system, we used text mining and data mining techniques to explore new intricacies of information embedded in the biomedical literature that we may not be aware of at first glance. By retrieving redox relating molecules from the DES‐RedoxVasc, we established new links and relationships between different components, such as with hydrogen peroxide and with Ang II. The results also suggest an overlapping interplay of processes that connect mitochondrial biogenesis and antioxidant systems with miR‐3178 and miR‐937. This shows that a cell expresses a quality control mechanism in the Redox homeostatic mechanisms of the vascular endothelial system. By using data mining in a review process, we have also deepened our understanding to our view the intricacies of how ROS is utilized in the vascular endothelial system redox system and also easier to see new pathways for improved treatment and more adequate prevention of cardiovascular‐related diseases.

## CONFLICT OF INTEREST

The authors declare no potential conflict of interest.
